# Dexmedetomidine attenuates neuronal injury after spinal cord ischaemia‐reperfusion injury by targeting the CNPY2‐endoplasmic reticulum stress signalling

**DOI:** 10.1111/jcmm.14688

**Published:** 2019-10-18

**Authors:** Lina Zhao, Meili Zhai, Xu Yang, Hongjie Guo, Ying Cao, Donghui Wang, Ping Li, Chong Liu

**Affiliations:** ^1^ Department of Anaesthesiology Tianjin Hospital Tianjin China; ^2^ Department of Anesthesiology Tianjin Central Hospital of Gynecology Obstetrics Gynecology Obstetrics Hospital of Nankai University Tianjin Key Laboratory of human development and reproductive regulation Tianjin China; ^3^ Department of medicine Tianjin Medical College Tianjin China; ^4^ Department of Critical Care Medicine Tianjin 4th Centre Hospital Tianjin China; ^5^ Department of Medical Imaging Shanxi Medical University Taiyuan City China; ^6^ Department of Anaesthesiology Central Laboratory Tianjin 4th Centre Hospital The Fourth Central Hospital Affiliated to Nankai University The Fourth Center Clinical College of Tianjin Medical University Tianjin China

**Keywords:** apoptosis, dexmedetomidine, endoplasmic reticulum, ischaemia‐reperfusion injury, spinal cord

## Abstract

Dexmedetomidine (Dex) has been proven to exert protective effects on multiple organs in response to ischaemia‐reperfusion injury, but the specific mechanism by which this occurs has not been fully elucidated. The purpose of this study was to investigate whether Dex attenuates spinal cord ischaemia‐reperfusion injury (SCIRI) by inhibiting endoplasmic reticulum stress (ERS). Our team established a model of SCIRI and utilized the endoplasmic reticulum agonist thapsigargin. Dex (25 g/kg) was intraperitoneally injected 30 minutes before spinal cord ischaemia. After 45 minutes of ischaemia, the spinal cord was reperfused for 24 hours. To evaluate the neuroprotective effect of Dex on SCIRI, neurological function scores were assessed in rats and apoptosis of spinal cord cells was determined by TUNEL staining. To determine whether the endoplasmic reticulum apoptosis pathway CNPY2‐PERK was involved in the neuroprotective mechanism of Dex, the expression levels of related proteins (CNPY2, GRP78, PERK, CHOP, caspase‐12, caspase‐9 and caspase‐3) were detected by western blot analysis and RT‐PCR. We observed that Dex significantly increased the neurological function scores after SCIRI and decreased apoptosis of spinal cord cells. The expression of ERS‐related apoptosis proteins was significantly increased by SCIRI but was significantly decreased in response to Dex administration. Taken together, the results of this study indicate that Dex may attenuate SCIRI by inhibiting the CNPY2‐ERS apoptotic pathway.

## 1 INTRODUCTION

After restoration of blood perfusion, original injury to ischaemic spinal cord tissue is aggravated, and even delayed death of spinal neurons occurs. This phenomenon is known as spinal cord ischaemia‐reperfusion injury (SCIRI), a common and serious secondary injury observed clinically. Spinal cord ischaemia‐reperfusion injury often occurs after surgery of the spine, spinal cord and thoracoabdominal aorta.^1^, ^2^ It has characteristics including disability, high mortality and poor prognosis and is a clinical problem that urgently needs to be solved. The pathogenesis of SCIRI is complex, and the specific mechanism of spinal cord death in response to reperfusion is not clear. Studies have shown that spinal cord ischaemia and reperfusion may be related to excitotoxicity, immunologic injury, disturbances in mitochondrion function and apoptosis.^3^, ^4^, ^5^, ^6^ Although significant progress has been made in the treatment of spinal ischaemic injury in recent years, SCIRI remains a major unresolved issue. Identifying new drugs or new methods to protect and save injured spinal cord tissue is important for treatment of this pathology.

In recent years, increasing literature has reported that endoplasmic reticulum stress (ERS) is involved in the occurrence of SCIRI.^7^, ^8^, ^9^, ^10^ Mild spinal cord ischaemia/reperfusion may lead to induction of the unfolded protein response (UPR) in the endoplasmic reticulum (ER). However, persistent and severe ERS activates the ERS‐related apoptosis pathway, which ultimately aggravates SCIRI.^7^, ^8^, ^9^, ^10^ Glucose‐regulated protein 78/immunoglobulin heavy chain‐binding protein (GRP78/BiP) is a marker protein of ERS. Under physiological conditions, GRP78 combines with three ER transmembrane proteins, protein kinase R‐like ER kinase (PERK), inositol‐requiring enzyme 1 (IRE1) and activating transcription factor 6 (ATF6), in an inactive state.^11^, ^12^ However, under pathological conditions, such as SCIRI, GRP78 dissociates from these transmembrane proteins, initiating relevant transmembrane protein signalling pathways and ultimately activating the three classical apoptotic pathways (CHOP, caspase‐12) mediated by ERS, leading to apoptosis.^13^, ^14^ However, it is unclear how the UPR sensor is activated. Recent studies have shown for the first time that ER‐localized protein homolog 2 (CNPY2) is involved in the UPR in the ER, and during transition from the non‐stressed state to the stressed state, the CNPY2 binding partner is converted from GRP78 to PERK, selectively initiating PERK‐CHOP‐mediated apoptosis signalling.^15^ CNPY2 can be detected in various rat tissues, including the nervous, respiratory, digestive and reproductive systems.^16^ CNPY2 enhances neurite outgrowth, which is critical for the development of the central nervous system.^16^ Therefore, CNPY2 may play a key role in the development of SCIRI.

Dexmedetomidine (Dex) is a novel and highly selective α2 adrenergic receptor agonist with sedative, anxiolytic, hypnotic, analgesic and sympatholytic effects, with reduced respiratory depression.^17^ Recently, increasing attention has been paid to Dex in clinical practice. At present, Dex is widely used in regional nerve blocks, general anaesthesia, ICU, paediatric sedation, in the emergency department and in neurosurgery.^18^ In addition to its anaesthetic characteristics, Dex was also shown to exert neuroprotective effects on SCIRI by inhibiting apoptosis or inflammation.^5^, ^19^, ^20^ However, the specific mechanism whereby this occurs has not yet been elucidated.

Based on these observations, ERS plays an important role in the development of SCIRI, and Dex alleviates SCIRI by inhibiting apoptosis and inflammation. Therefore, the purpose of this study was to investigate whether Dex attenuates SCIRI by inhibiting ERS. In addition, we investigated whether CNPY2 is involved in the PERK‐CHOP apoptotic pathway in response to SCIRI.

## 2 MATERIALS AND METHODS

### 2.1 Animals

Healthy male Sprague‐Dawley rats (SD, 14‐16 weeks, 280‐350 g) were provided by the Institute of Zoology, Chinese Academy of Medical Sciences. All experimental schemes and animal treatment procedures were performed according to the guidelines of the Tianjin Hospital Experimental Animal Ethics Committee. All rats were housed at a constant temperature of 22‐25°, 50%‐60% relative humidity and 12‐hour light/12‐hour dark circulation environment with free access to food and water. All rats were acclimatized to their environment for 1 week before the experiment.

### 2.2 Materials

Dex was purchased from Jiangsu Enhua Pharmaceutical Co., Ltd., National Pharmaceutical Standard H20110086.

### 2.3 Experimental groupings and drug treatment

All healthy male rats were randomly divided into four groups: sham operation + saline group (S group, n = 18), which received intraperitoneal injection of normal saline 30 minutes before the surgery, which only received abdominal aorta separation with no clip closed; Dex alone group (Dex group, n = 18), which received Dex (25 µg/kg) intraperitoneally injected 30 minutes before the surgery with the same operation as the S group; ischaemia/reperfusion + saline group (I/R group, n = 18), which received intraperitoneal injection of normal saline 30 minutes before the surgery, spinal cord ischaemia for 45 minutes and reperfusion for 24 hours; ischaemia/reperfusion + Dex group (I/R + Dex group, n = 18), which received intraperitoneal injection of Dex (25 µg/kg) 30 minutes before surgery, spinal cord ischaemia for 45 minutes and reperfusion for 24 hours. According to previous studies, a single intraperitoneal injection of Dex (25 μg/kg) was selected to study the neuroprotective effect of SCIRI.^2^, ^21^, ^22^ S and I/R groups were given equal volume of saline.

Secondly, to better explain the possible protective mechanism of Dex on SCIRI, we added an ER agonist thapsigargin (TG, 3 mg/kg, soluble in DMSO, Abcam) in later experiments as previously described.^23^, ^24^, ^25^ The experiment was performed as follows (n = 6): I/R + DMSO group (I/R + D), which received intraperitoneal injection of the same volume of DMSO 30 minutes before surgery, followed by SCIRI surgery; I/R + DMSO+Dex group (I/R + D + Dex group), which received the same volume of DMSO and Dex (25 μg/kg) intraperitoneally injected 30 minutes before surgery, followed by SCIRI; I/R + TG group (I/R + TG), which received intraperitoneal injection of TG (3 mg/kg) 30 minutes before surgery followed by SCIRI operation; I/R + TG + Dex group, which received TG (3 mg/kg) and Dex (25 μg/kg) intraperitoneally injected 30 minutes before surgery, and then SCIRI operation.

### 2.4 Preparation of the SCIRI model

The SCIRI model was established according to the Zivin method.^26^ Rats were fasted for 8 hours before surgery. Briefly, abdominal anaesthesia was administered with 2% sodium pentobarbital (40 mg/kg). The abdominal cavity was exposed to the abdominal aorta in strict accordance with aseptic procedures. The bilateral renal arteries were isolated. Next, the abdominal aorta was clamped between the left and right renal arteries with a non‐invasive artery clamp, and it was confirmed that the abdominal aorta pulsation disappeared after the arterial clip disappeared. After clamping for 45 minutes, the arterial clamp was loosened to allow reperfusion. The peritoneal cavity was closed after ensuring that no bleeding or damage to the arteries had occurred. The body temperature of rats was kept constant throughout the procedure. All animals were awake within 60 minutes after surgery. Neurological behavioural scores were assessed 24 hours after SCIRI in rats. Subsequently, three rats in each subgroup were euthanized to obtain lumbar spinal cord tissue, which was fixed in 4% paraformaldehyde, embedded in paraffin and cut into spinal cord sections for HE and TUNEL staining. Lumbar spinal cord tissue was immediately collected from the other three rats in each group and was stored at −80°C until western blot and RT‐PCR detection.

### 2.5 Neurological function score

Twenty‐four hours after SCIRI, rats were removed from their cages and placed in an open field. Motor function of the hindlimbs was recorded according to Basso, Beattie and Bresnahan (BBB) scoring criteria.^27^, ^28^ According to the presence of motor function defects, assigned scores range from 0 to 21 points. Hindlimbs were completely paralysed with a score of 0 points. For scores within the range of 1‐7 points, the hindlimb joints can only move within certain degrees. When the hindlimbs are capable of movement, the values range from 8 to 13 points according to gait and coordination. When claws can be used for fine movements, scores range from 14 to 20 points, and motor function is completely normal at 21 points. Scores were assigned by the same observer who was blinded to the specific grouping of scored rats.

### 2.6 TUNEL staining

Twenty‐four hours after ischaemia‐reperfusion, apoptosis of rat spinal cord cells was detected using terminal deoxynucleotide transferase‐mediated dUTP notch terminal labelling (TUNEL). According to the manufacturer's protocol, paraffin sections of the spinal cord were stained with the in situ cell apoptosis detection kit (POD; Roche Diagnostics Corp.). Five fields were randomly selected from each spinal cord section under light microscopy (400×), and the number of cells in the field of view relative to the number of apoptotic cells was quantified. The cell apoptosis rate was calculated as the apoptotic cell number/total cell number × 100%

### 2.7 Western blot analysis

#### 2.7.1 Extraction of total cell lysate from spinal cord samples

Twenty‐four hours after SCIRI, frozen spinal cord specimens were used for western blot analysis. Protein separation and western blotting were performed as previously described.^29^, ^30^ Briefly, spinal cord specimens were lysed in 100 μL ice‐cold RIPA lysis buffer (Solarbio) supplemented with 1 μL of 100 mmol/L phenylmethylsulfonyl fluoride (Sigma) for 30 minutes. Tissue homogenates were centrifuged at 12 000 *g* for 30 minutes at 4°C, and the supernatant was taken as the total cell lysate.

#### 2.7.2 Extraction of spinal cord ER

After full lysis of spinal cord specimens for 30 minutes, tissue homogenates were centrifuged (4°C, 800 *g*) for 10 minutes, and supernatants were centrifuged (4°C, 10 000 *g*) for 20 minutes. The new supernatants were collected again for centrifugation (4°C, 100 000 *g*) for 60 minutes, and the sediment was the ER. A lysate containing 1% Triton X‐100 was used to suspend ER samples.

#### 2.7.3 Isolation of target proteins

The total protein and ER protein lysates were quantified using the Bradford method. Protein samples (50 μg/lane) were electrophoresed on 10% or 12% SDS‐polyacrylamide gels and transferred to polyvinylidene fluoride membranes (PVDF, Millipore Corporation). PVDF membranes were blocked with 5% skim milk blocking solution for 1 hour, and then the appropriate dilution ratio of primary antibody was added and incubated overnight at 4°C. Then, membranes were washed three times and incubated with horseradish peroxidase‐conjugated secondary antibodies (1:10 000, Solarbio) for 1 hour at room temperature. Target bands were visualized using enhanced chemiluminescence (ECL, Merck Millipore) methods. Quantification of blots was performed using ImageJ software (Bio‐Rad). Calnexin and GAPDH were used as internal controls. All band intensities were standardized to GAPDH or Calnexin and are expressed as a percentage of the control group. Primary antibodies of the target proteins are as follows: CNPY2 (1:1000) from Proteintech; PERK (1:1000) and p‐PERK (1:1000) from Cell Signaling Technology; GRP78 (1:2000), caspase‐12 (1:10 000), ATF4 (1:5000), CHOP (1:2000), caspase‐ 9 (1:1000), Bcl‐2 (1:500), caspase‐3 (1:10 000), GAPDH (1:5000) and Calnexin (1:250) from Abcam.

### 2.8 RT‐PCR analysis

According to the manufacturer's instructions, total RNA was isolated from spinal cord specimens using the Rneasy Mini Kit (QIAGEN). Total RNA (4 μg) was reverse transcribed into cDNA using the PrimeScriptTM RT‐PCR Kit (TAKARA). Amplification conditions were as follows: 95°C for 2 minutes, 95°C for 15 seconds, 63°C for 30 seconds, extension 68°C for 1 minute and 25 cycles and finally 72°C for 4 minutes. PCR primers were as follows: CNPY2 (forward: 5′‐GTTTGCGTGTGAGAGCATTGT‐3′, reverse 5′‐ATCATGAGATCTGTGCAGGG‐3′), GRP78 (forward: 5′‐TGACAACGACAAGACCCCAC‐3′, reverse: 5′‐ GTCAATGCCAACCACCATGC‐3′), and Calnexin (5′‐GCCCAAAACGGGGAAGTTTT‐3′, reverse 5′‐AGGTACCCCAGACTTTTGCC‐3′). Data were standardized to Calnexin gene expression and analysed using Image Lab software (Bio‐rad, USA).

### 2.9 Statistical analysis

All data are expressed as the mean ± standard deviation, and comparisons among multiple groups were assessed by one‐way analysis of variance (ANOVA). In this experiment, SPSS13.0 statistical software was used for data processing. When differences were considered statistically significant, the least significant difference test was used to compare differences between two groups. For all data, *P* < .05 was considered to be statistically significant. All experiments in this study were performed in triplicate.

## 3 RESULTS

### 3.1 DEX pre‐treatment preserves neurological function after SCIRI

**Figure 1:**
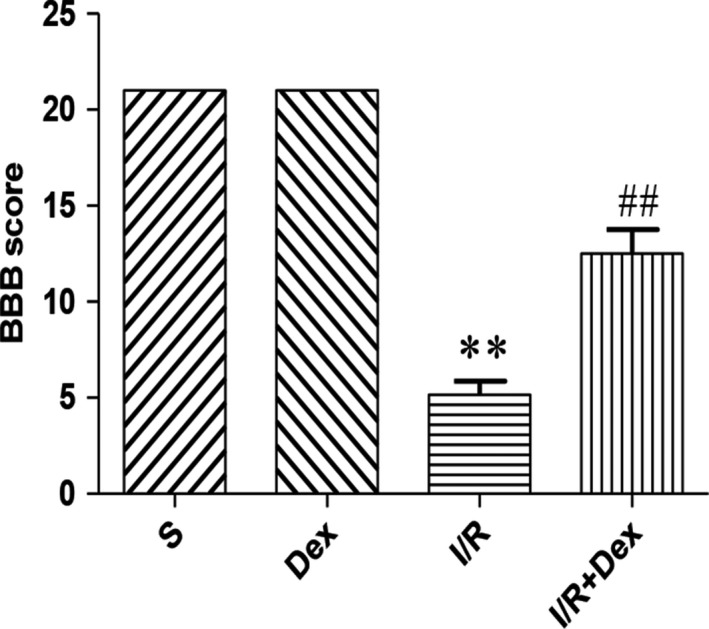
Dex pre‐treatment preserves neurological function after SCIRI. Data are shown as the mean ± SEM, n = 18 per group. *P < .05, **P < .01 compared with the S group; ^#^ *P* < .05, ^##^ *P* < .01 compared with the I/R group

To investigate whether SCIRI‐induced neurological deficits were altered by pre‐treatment with Dex, we used BBB scores to assess motor function in rats. No obvious neurological deficits were observed between the Dex and S groups, and there was no significant difference between the two groups. However, BBB score in the I/R group was significantly decreased compared with the S group. In contrast, compared with the IR group, the BBB score of rats in the I/R + Dex group was significantly increased (Figure 1). The results demonstrated that SCIRI causes neurological deterioration, and administration of Dex gradually improved functional outcomes of locomotor activity.

### 3.2 Dex pre‐treatment effectively attenuates spinal cord neuronal apoptosis after SCIRI

**Figure 2:**
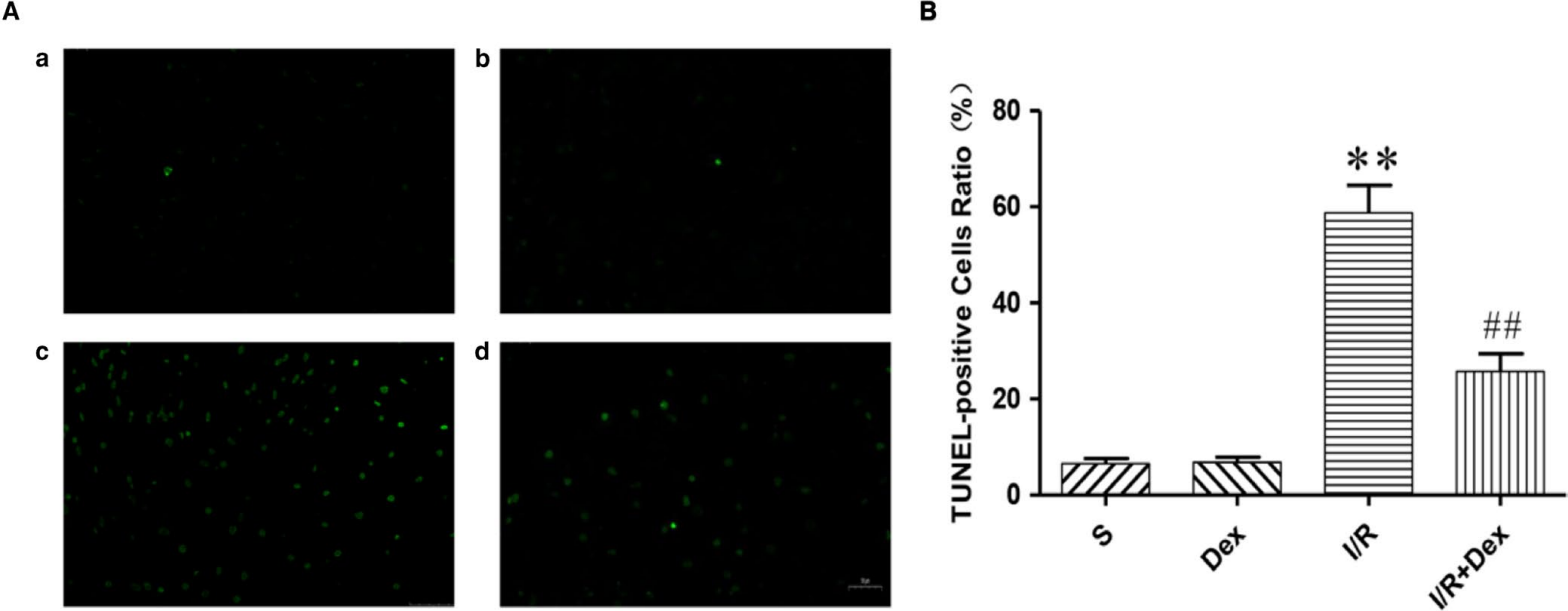
Dex pre‐treatment effectively attenuates spinal cord neuronal apoptosis after SCIRI. A, TUNEL staining was performed on slices from spinal cord specimens. B, Ratio of positive cells. Original magnification is 400×. n = 6. Data are shown as the mean ± SEM. *P < .05, *P < .05, **P < .01 compared with the S group; ^#^ *P* < .05, ^##^ *P* < .01 compared with the I/R group

To assess the effect of Dex on apoptosis in response to SCIRI, we used TUNEL assays, as shown in Figure 2. TUNEL‐positive cells were nearly undetectable in the S and Dex groups. In contrast, the proportion of TUNEL‐positive neurons was significantly increased in the I/R group, whereas the number of TUNEL‐positive neurons was significantly decreased in response to Dex treatment. These results confirm that Dex significantly inhibits apoptosis of spinal cord neurons after SCIRI.

### 3.3 Dex pre‐treatment effectively inhibits activation of ERS after SCIRI

**Figure 3:**
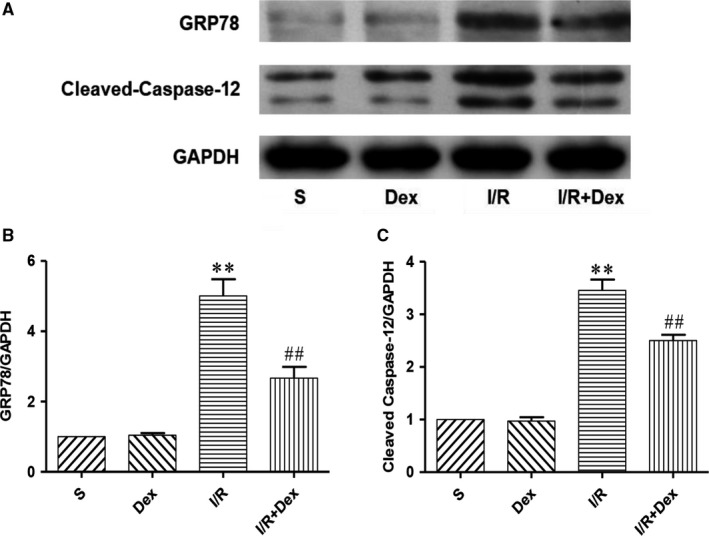
Dex pre‐treatment effectively inhibits activation of ERS after SCIRI. Expression of GAPDH was used as a loading control. The results were normalized to GAPDH expression. n = 6. Data are shown as the mean ± SEM. *P < .05, *P < .05, **P < .01 compared with the S group; ^#^ *P* < .05, ^##^ *P* < .01 compared with the I/R group

**Figure 4:**
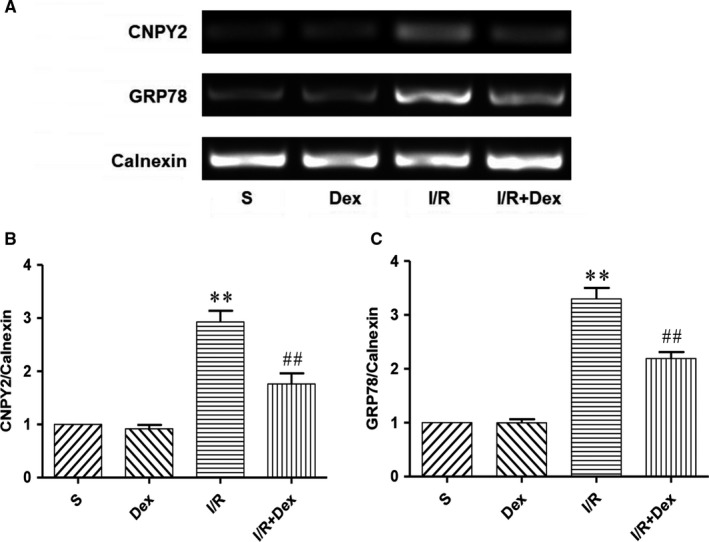
Dex pre‐treatment effectively attenuates the expression level of CNPY2 and GRP78 mRNA on ER of spinal cord induced by SCIRI. Expression of CNPY2 and GRP78 mRNA on ER was detected by RT‐PCR. Expression of Calnexin was used as a loading control. The results were normalized to Calnexin expression. n = 6. Data are shown as the mean ± SEM. *P < .05, *P < .05, **P < .01 compared with the S group; ^#^ *P* < .05, ^##^ *P* < .01 compared with the I/R group

To further determine whether ERS is involved in apoptosis in response to spinal cord injury, we measured expression of ERS marker proteins GRR78 and caspase 12 by western blot. Our results showed no change in protein expression in Dex and S groups, but expression levels of GRR78 and caspase‐12 in the I/R group were significantly increased compared with the S group (Figure 3). However, compared with the I/R group, expression of GRR78 and caspase‐12 in the I/R + Dex group was significantly decreased. Furthermore, we measured expression levels of GRP78 mRNA by RT‐PCR. The results showed that levels of GRP78 mRNA were consistent with western blotting results (Figure 4C). These experimental results suggest that ERS is involved in the pathogenesis of SCIRI, and administration of Dex significantly inhibits ERS.

### 3.4 The effect of Dex on expression of ERS‐dependent apoptotic proteins after SCIRI

**Figure 5:**
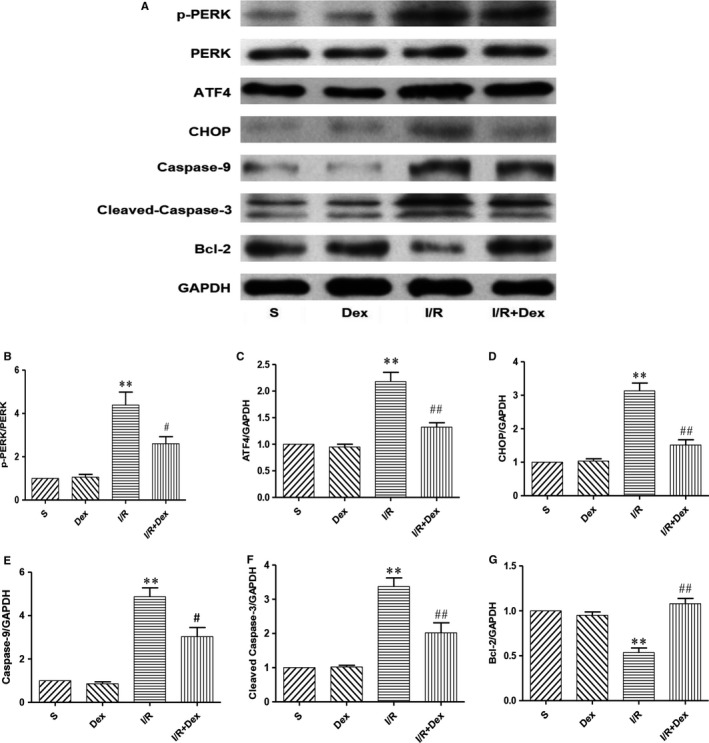
Effect of Dex on the expression of ERS‐dependent apoptotic protein after SCIRI. Expression of GAPDH was used for the loading control. The results were normalized to percentage of GAPDH expression. n = 6. Data are shown as mean ± SEM. *P < .05, *P < .05, **P < .01 compared with the S group; ^#^ *P* < .05, ^##^ *P* < .01 compared with the I/R group

To test whether spinal cord ER is involved in the survival effects of Dex treatment, we examined the expression of ERS‐mediated apoptotic signalling proteins, ATF4, CHOP, caspase‐9 and caspase‐3. Western blot results revealed that spinal cord ischaemia‐reperfusion significantly increased expression levels of ERS‐related apoptotic proteins ATF4, CHOP, caspase‐9 and caspase‐3 compared with the S group. Compared with the I/R group, protein expression of ATF4, CHOP, caspase‐9 and caspase‐3 in the I/R + Dex group was significantly decreased (Figure 5). However, Bcl‐2 results were reversed. Expression of Bcl‐2 protein in the I/R group was significantly decreased, while being significantly increased in the I/R + Dex group (Figure 5). Taken together, our data suggest that the ERS‐mediated apoptosis signalling pathway may be involved in the induction of apoptosis after SCIRI, and the protective effect of Dex may partly be induced by inhibiting apoptosis.

### 3.5 Dex pre‐treatment effectively attenuates expression of CNPY2 induced by SCIRI

**Figure 6:**
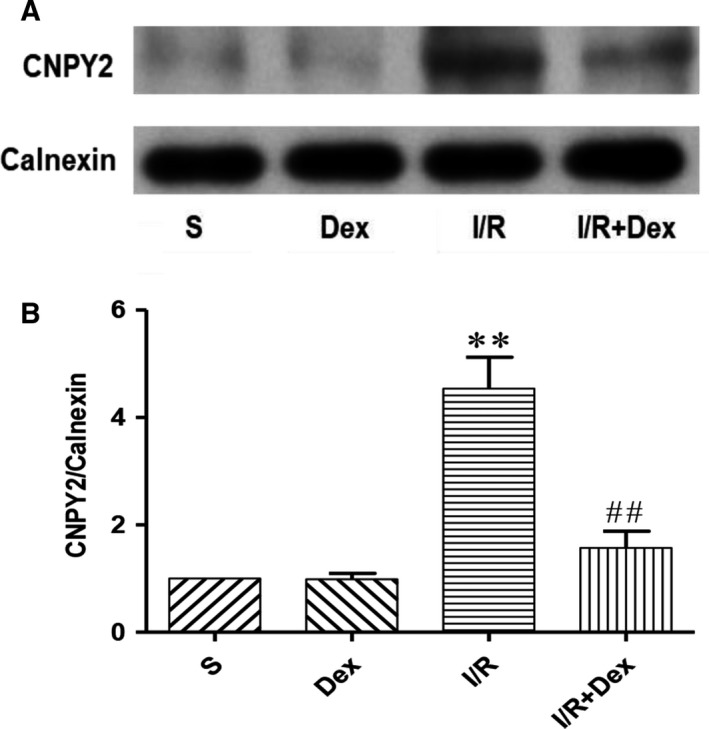
Dex pre‐treatment effectively attenuates expression of CNPY2 induced by SCIRI. Expression of CNPY2 on ER in spinal cord was detected by western blot. Expression of Calnexin was used as a loading control. The results were normalized to Calnexin expression. n = 6. Data are shown as the mean ± SEM. *P < .05, *P < .05, **P < .01 compared with the S group; ^#^ *P* < .05, ^##^ *P* < .01 compared with the I/R group

Studies have shown that CNPY2 plays a key role in ERS and is involved in the development of many diseases, including metabolic disorders and inflammation. CNPY2 is released from the ER and then activates the PERK‐CHOP pathway of the ER apoptotic pathway, initiating UPR. To determine whether CNPY2 is involved in the development of ERS after spinal cord injury, we examined expression of CNPY2 in spinal cord ER. As shown in Figures 4 and 6, RT‐PCR and western blot results were not statistically different with respect to expression of CNPY2 between S and Dex groups. Compared with the S group, expression of CNPY2 was significantly increased in the I/R group. In addition, Dex significantly inhibited CNPY2 expression. These results suggest that CNPY2 is also expressed in the spinal cord and is involved in the development of SCIRI. Administration of Dex significantly inhibited expression of CNPY2, further demonstrating that Dex may protect the spinal cord by inhibiting the ERS pathway.

### 3.6 Dex pre‐treatment effectively inhibits the CNPY2‐PERK–dependent apoptosis signalling pathway after SCIRI

To confirm whether Dex protects the spinal cord from ischaemia‐reperfusion injury by inhibiting the CNPY2‐PERK–mediated apoptotic signalling pathway, we examined changes in the ER transmembrane protein PERK (Figure 5B). Expression levels of PERK protein in the S and Dex alone groups were not statistically different. In the I/R group, expression levels of PERK protein were significantly increased, while in the I/R + Dex group, PERK was significantly decreased. These results demonstrate that Dex protects the spinal cord from ischaemia‐reperfusion injury by inhibiting expression of the transmembrane protein PERK in ER.

**Figure 7:**
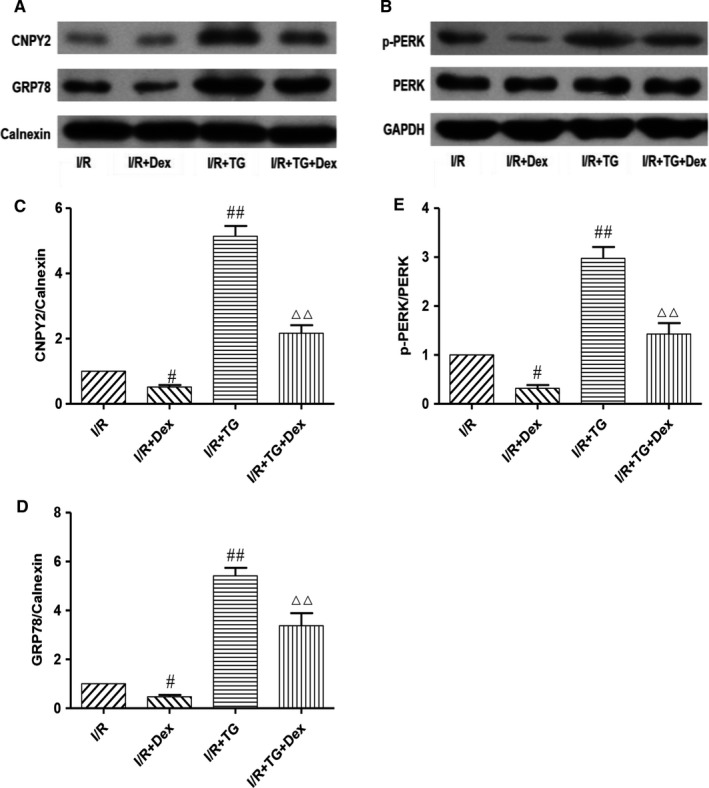
Dex pre‐treatment effectively inhibits CNPY2‐PERK–dependent apoptosis signalling pathway induced by ER agonists after SCIRI. Expression levels of CNPY2 (C), GRP78(D), and p‐PERK (E) proteins in spinal cord samples were detected by western blot (A, B). Expression of Calnexin or GAPDH was used as a loading control. The results were normalized to Calnexin or GAPDH. n = 6. Data are shown as the mean ± SEM. ^#^ *P* < .05, ^##^ *P* < .01 compared with the I/R group; ^△^ *P* < .05, ^△△^ *P* < .01 compared with the I/R + TG group

To further determine whether Dex protects the spinal cord from ischaemia‐reperfusion injury by inhibiting CNPY2‐PERK mediated apoptosis, we introduced an ER agonist and assessed changes in ERS‐related apoptotic proteins. As shown in Figure 7, protein expression levels of CNPY2 in the I/R + TG group were significantly higher than in the I/R group, but expression levels of CNPY2 were significantly decreased in response to Dex treatment. In addition, Dex significantly inhibited expression levels of GRP78 and p‐PERK induced by TG after SCIRI (*P* < .01). The above results indicate that Dex attenuates spinal cord injury from ischaemia‐reperfusion in part by inhibiting the CNPY2‐PERK pathway.

## 4 DISCUSSION

Owing to segmental blood supply and relatively poor collateral circulation, the spinal cord is prone to ischaemia‐reperfusion injury. Spinal cord ischaemia‐reperfusion injury can lead to dysfunction and lifelong disability, seriously affecting patient quality of life and increasing the heavy burden to family and society. Spinal cord ischaemia‐reperfusion injury involves a series of complex pathophysiological mechanisms, including excitatory neurotransmitter injury, oxidative stress, inflammatory response, neuronal apoptosis and autophagy.^4^ Many studies previously confirmed that SCIRI causes neuronal apoptosis, resulting in neurological dysfunction. However, the mechanism whereby this occurs has not yet been elucidated and remains an unresolved issue in medical research. Therefore, our goal was to explore potential signalling pathways involved in SCIRI. Understanding these mechanisms is crucial for identifying reliable protective treatment strategies. In this study, our data indicated that CNPY2 is expressed in the spinal cord and participates in SCIRI. Administration of Dex significantly inhibited expression of CNPY2 and ERS‐induced neuronal damage by inhibiting the CNPY2‐PERK pathway. This treatment strategy weakened the effects of SCIRI and provided an extended therapeutic window for neuroprotection.

Spinal cord neurons are highly sensitive to various injury factors such as ischaemia and hypoxia.^31^ The pathological mechanism of SCIRI is very complex and has not yet been entirely elucidated. Many studies have shown that transient ischaemia and hypoxia can cause spinal nerve necrosis.^31^ The primary mechanisms whereby this occurs include free radical and lipid peroxidation, intracellular calcium disruption, release of inflammatory factors, excitatory neurotransmitter damage, apoptosis of nerve cells and a series of pathophysiological processes.^3^, ^4^, ^5^, ^6^ Continual ischaemia and hypoxia can aggravate SCIRI, resulting in secondary oedema of the spinal cord and increased intrathecal pressure, potentially leading to increased tissue damage and aggravated loss of motor function. At present, in the study of SCIRI, determination of rat neurological function injury primarily uses hindlimb movement function BBB scores. The results of this experiment confirmed that SCIRI leads to hindlimb motor dysfunction, which exacerbates spinal motor injury.

Spinal cord ischaemia can cause irreversible metabolic, neuronal structure and function disorders, eventually leading to cell death. Cell death is primarily divided into two major types, necrosis and apoptosis.^32^ Necrosis is the death of local tissue cells characterized by changes in enzyme solubility. Primary manifestations include cell swelling, organelle disintegration and protein denaturation.^33^ Apoptosis is an active death mode of cells that plays an important role in maintaining stability of the internal environment.^34^ Morphological characteristics of apoptosis include cell shrinkage, compact cytoplasm and formation of apoptotic bodies.^34^ Apoptosis does not cause an inflammatory response in surrounding tissues. The mechanism of apoptosis in SCIRIR has attracted increasing attention. Apoptosis is not caused directly by spinal cord injury; instead, it is a major cause of cell death in secondary spinal cord injury, which plays an important role in neurological function after injury. To further verify our experimental results, we assessed apoptosis in spinal cord cells using the TUNEL method. Experimental results confirmed that SCIRI significantly aggravates necrosis of neurons and significantly aggravates apoptosis.

As a new type of anaesthesia adjuvant, Dex has pharmacological characteristics, such as anxiolytic, sedative, analgesic and anti‐sympathetic activity, without obvious respiratory inhibition.^17^ Therefore, clinically, Dex is widely used in sedation and analgesia during perioperative period and ICU. More and more studies show that Dex has strong anti‐apoptotic and anti‐inflammatory effects in addition to its anaesthetic characteristics.^5^, ^19^, ^20^ Dex reduces ischaemia‐reperfusion injury in the myocardium, brain, kidney and lung by exerting anti‐apoptotic and anti‐inflammatory effects.^35^, ^36^, ^37^, ^38^ Consistent with previous studies, our experiments found that Dex significantly improves motor dysfunction caused by SCIRI. TUNEL results also confirmed that use of Dex significantly inhibited apoptosis induced by SCIRI. However, its protective mechanism against neuronal apoptosis after SCIRI has not been clarified.

At present, domestic and foreign studies have shown that one of the mechanisms of pathogenesis in SCIRI is the disturbance of calcium balance, including extracellular calcium influx, increase of calcium release from the ER and eventual intracellular calcium overload.^39^, ^40^ Intracellular calcium homeostasis is primarily maintained by the ER and plays an important role in spinal cord injury after ischaemia/reperfusion.^4^ Mild tissue ischaemia/reperfusion can lead to ER dysfunction and induces the unfolded protein response of the ER, thereby relieving cellular stress and protecting cells from apoptosis or necrosis. However, persistent and severe ERS exceeds the ability of the ER to handle unfolded proteins, eventually triggering the ERS apoptotic pathway.^41^ The ER chaperone protein GRP78/BIP (glucose regulatory protein 78/immunoglobulin heavy chain‐binding protein) is primarily present in the ER and is a marker of ERS. Under physiological conditions, the ER chaperone GRP78 interacts with three ER transmembrane proteins to form a stable complex, which inhibits its activity. However, under pathological conditions, such as spinal IRI, expression levels of the GRP78 protein significantly increase, triggering ERS and subsequent induction of ERS‐associated gene expression for factors such as CHOP and caspase‐12, eventually leading to apoptosis.^41^, ^42^ In this study, the expression levels of GRP78 mRNA and GRP78 protein were markedly increased after spinal cord I/R, and the protein expression level of caspase‐12 was significantly increased. Endoplasmic reticulum agonists also significantly increased expression of CNPY2 after SCIRI. After administration of Dex, expression levels of GRP78 mRNA and protein were significantly decreased, and the protein expression levels of caspase‐12 were also decreased. Dex also reduced protein expression of GRP78 induced by ER agonists after SCIRI. These results demonstrate that Dex protects the spinal cord from ischaemia‐reperfusion injury by inhibiting the ERS pathway.

Ischaemia‐reperfusion injury induces UPR, subsequently leading to dissociation of the ER molecular chaperone GRP78 from three ER transmembrane proteins and inducing ER apoptosis.^41^ However, it is not fully understood how the UPR pathway is triggered. Recent studies have found that CNPY2 is involved in UPR and is a major trigger of the PERK‐CHOP pathway. During transformation from the non‐stressed to the stress state of the ER, the CNPY2 binding partner is converted from GRP78 to PERK, selectively activating PERK‐CHOP–mediated apoptosis signalling pathways.^15^, ^43^ Some authors also produced CNPY2‐deficient cells using gene knockout techniques and observed nearly complete inhibition of PERK activation, suggesting that CNPY2 expression is a key step of signal initiation.^15^ CNPY2 belongs to the CNPY family and is widely distributed in various rat tissues, including the nervous, respiratory, digestive and reproductive systems.^16^ However, the known role of CNPY2 is limited. It has been reported that CNPY2 enhances neurite outgrowth, which is crucial for development of the central nervous system.^16^ We experimentally confirmed the involvement of ERS in the development of SCIRI. Therefore, we hypothesized that CNPY2 is also distributed in the spinal cord and is involved in the development of SCIRI. We purified spinal cord ER and detected CNPY2 mRNA and protein expression, and expression was significantly increased in response to SCIRI. Our experiments confirmed that ERS is involved in the development of SCIRI. Therefore, we hypothesized that CNPY2 is also distributed in the spinal cord and is involved in the development of SCIRI. We purified spinal cord ER and measured CNPY2 mRNA and protein expression. Levels of CNPY2 mRNA and protein were significantly increased after ischaemia‐reperfusion, and ER agonists significantly increased expression of CNPY2 after SCIRI. In this study, Dex treatment attenuated expression of CNPY2 in ischaemia‐reperfusion rats and reduced the expression of CNPY2 induced by ER agonists after SCIRI. These results confirm that CNPY2 is present in the spinal cord and is involved in ERS induced by SCIRI. Furthermore, Dex may play an important role in inhibiting neuronal apoptosis induced by ERS by inhibiting CNPY2.

Recent studies have shown that CNPY2 specifically regulates PERK, which is a key factor in initiating PERK‐CHOP–mediated UPR.^15^, ^43^ More importantly, the study found that a lack of CNPY2 did not affect IRE1 signal transduction. To gain a deeper understanding of the role of CNPY2 in ER stress, we examined proteins associated with the apoptosis pathway of PERK‐CHOP. Our results confirmed that SCIRI results in significantly increased protein expression of PERK, CHOP and caspase‐3. Protein expression of Bcl‐2 was the opposite. Dex treatment weakened expression of CNPY2 and attenuated the pro‐apoptotic factors PERK, CHOP and caspase‐3 protein expression, increasing expression of the Bcl‐2 protein. In addition, Dex treatment reduced expression of p‐PERK by ER agonists after SCIRI. Combined with the above experimental results, we speculate that the CNPY2‐PERK pathway participates in the pathogenesis of SCIRI, and administration of Dex protects tissues from SCIRI by inhibiting this pathway.

In this study, our data demonstrated that CNPY2 is expressed in the spinal cord and participates in SCIRI. Dex reduced expression of GRP78 and caspase‐12 after ischaemia/reperfusion injury, indicating that Dex attenuates ER stress. In addition, we found that Dex attenuates expression of CHOP and caspase‐3 through CNPY2‐PERK signalling, suggesting that Dex may inhibit ER stress‐induced neuronal injury by inhibiting the CNPY2‐PERK apoptotic pathway. However, the pathological process of ischaemia‐reperfusion injury involves a variety of complex mechanisms, and the primary mechanism is not yet widely agreed upon. In addition, it is unclear whether Dex alleviates SCIRI by inhibiting inflammation, autophagy or other signalling pathways. Therefore, we will continue to study the mechanism of neuronal apoptosis and the optimal drug target for the prevention and treatment of apoptosis in the process of ischaemic reperfusion in the spinal cord to accelerate rehabilitation after ischaemia‐reperfusion injury.

## CONFLICT OF INTEREST

The authors declare no conflict of interest.

## AUTHOR CONTRIBUTIONS

CL, LNZ and MLZ designed the experiments. LNZ, MLZ and XY performed the experiments. HJG, YC and DHW analysed the data. CL, LNZ and MLZ wrote the manuscript. CL, LMZ and PL edited the manuscript. All authors read and approved the final manuscript.
